# Double burden of malnutrition in Afghanistan: Secondary analysis of a national survey

**DOI:** 10.1371/journal.pone.0284952

**Published:** 2023-05-30

**Authors:** Obaidullah Fahim, Shafiqullah Shahim, Ahmad Nawid Shams, Ahmad Farshid Muhammadi, Abolghassem Djazayery, Ahmad Esmaillzadeh

**Affiliations:** 1 Department of Community Nutrition, School of Nutritional Sciences and Dietetics, Tehran University of Medical Sciences, Tehran, Iran; 2 Faculty of Public Health, Department of Nutrition, Kabul University of Medical Science, Kabul, Afghanistan; 3 Faculty of Public Health, Department of Health Management and Policy, Kabul University of Medical Science, Kabul, Afghanistan; 4 Department of Health Management Information System, Ministry of Public Health, Kabul, Afghanistan; 5 Postgratuate Medical Education Directorate (PGME), Ministry of Public Health, Kabul, Afghanistan; 6 Obesity and Eating Habits Research Center, Endocrinology and Metabolism Molecular -Cellular Sciences Institute, Tehran University of Medical Sciences, Tehran, Iran; 7 Department of Community Nutrition, School of Nutrition and Food Science, Isfahan University of Medical Sciences, Isfahan, Iran; MRC Unit The Gambia at LSHTM, GAMBIA

## Abstract

**Background:**

Reports about the magnitude of co-existence of under- and over-nutrition is limited in Afghanistan. This study aimed to assess the prevalence of double burden of malnutrition (DBM) at individual and household level in Afghanistan.

**Methods:**

This study was done based on the Afghanistan National Nutrition Survey 2013, which included a representative sample of 126,890 individuals (including more than 18,000 households) throughout Afghanistan. Intra-individual DBM was defined as the co-existence of “overweight/obese” and “stunting or micronutrient deficiencies” (including anemia, vitamin A deficiency, vitamin D deficiency and iodine deficiency). At the household level, DBM was considered as having at least one household member as overweight/obese and at least one another member of that household as undernourished (stunted, wasted, underweight or any micronutrient deficiency). SPSS and Stata software were used in the current analysis. Cross-tabulations was used to estimate the prevalence and its 95% confidence interval(CI). This study was ethically approved at Tehran University of Medical sciences.

**Results:**

The overall prevalence of intra-individual DBM was 12.5% (95% CI: 12.1; 12.9). Among the whole study participants at individual level of DBM, 11.7% (11.3; 12.1) of individuals had overweight along with stunting simultaneously and 20.5% (18.8; 22.4) had overweight and micronutrient deficiencies at the same time at individual level. The household level of DBM was found among 28.6% (95% CI: 27.9; 29.4) of households; such that 27.3% (26.6; 28.1) of households had at least one member with overweight and another member with stunting or wasting or underweight. Co-existence of overweight and micronutrient deficiencies at the same household was seen in 38.3% (35.5; 41.2).

**Conclusion:**

This study demonstrated a high prevalence of DBM at individual and household level in Afghanistan. Therefore, developing appropriate national macro-policies and strategies and designing appropriate programs such as public awareness programs, subsidization, food assistance programs, food fortification and dietary supplementation should be implemented by the ministry of public health, inter- related organs and international health agencies to reduce the burden of this problem in this country.

## Introduction

Double burden of malnutrition (DBM), a complex phenomenon, is co-occurrence of over nutrition along with under-nutrition within the same individual, household, community or population [1–4]. Both sides of malnutrition are equally harmful to human health and impose a great burden to the healthcare system. Nutrition deficit prevents physical and intellectual development, while nutrition excess contributes significantly to various non-communicable diseases [5–8].

The phenomenon of DBM is prevalent in developing countries due to the nutrition transition occurring in these regions [9]. Therefore, the rate of overweight/obesity and related chronic disease are increasing, though under nutrition still remains prevalent in these countries [9–11]. Based on data from WHO in 2014, 1.9 billion adults were affected by overweight or obesity, at the same time, 462 million were underweight worldwide [12]. Globally, the number of undernourished people raised from 777 million in 2015 to 815 million in 2016. Simultaneously, adult obesity is rising in all regions [13].

Several studies have revealed magnitude of DBM at different regions and countries [14–16]. National surveys from Vietnam, Brazil, Russia, Indonesia, Kyrgyzstan, China and USA reported the prevalence of DBM ranging from 3.7 to 15.5% of households [14]. Based on data from 42 developing countries in Africa, Asia and Latin America, it has been shown that 0.9 to16.0% of the households had a stunted child and an overweight or obese mother [15]. The prevalence of household DBM was estimated to be 4.10% in Bangladesh, 1.54% in Nepal, 3.93% in Pakistan and 5.54% in Myanmar in 2019 [16].

Despite some reports from Afghanistan about the health status of Afghan people, limited information is available about the nutritional status of this population. The available information in this regard is mostly confined to general nutrition status and nutrient deficiencies in different age groups. For instance, stunting, wasting and overweight is prevalent among 40.9%, 9.5% and 5.4%, respectively, of children aged under 5 y [17]. However, we are aware of no data about the DBM among Afghan people at individual, household and national levels. Given the increased urbanization in Afghanistan in recent years along with nutrition transition in this region, assessment of double burden of malnutrition might provide appropriate information for policy makers in the country to battle the condition. Therefore, the objective of the present study was to examine the prevalence of DBM in Afghani people at the individual and household levels. This estimation was done based on data from the National Nutrition Survey in Afghanistan [17].

## Participants and methods

### Study population

This study was done based on data from National Nutrition Survey of Afghanistan, which was done at 2013 [17]. The survey was a cross-sectional study at household level across Afghanistan. Sample size was calculated to provide national and provincial representative estimates. The survey was conducted in all 34 provinces of Afghanistan targeting 18,360 households. A stratified two-stage cluster sampling method was adopted in the survey to recruit households. Each of 34 provinces were considered as an independent stratum. Enumeration areas at each province were primary sampling units (PSU) and 30 enumeration areas were selected from each province. Households within each primary sampling units were secondary sampling unit (SSU) and 18 households were selected from each PSU. Among 18,360 households, data were not collected for 310 households due to security concerns, duplication, and inaccessibility problems. Finally, the whole number of households assessed in Afghanistan National Nutrition Survey was 18,050 households. Description of survey population is given in **S1 Table.**

The national nutrition survey design, sampling strategy, instruments and analytical plans have been reviewed and approved by the Institutional Review Board (IRB) of Ministry of Public Health, Government of Afghanistan. The survey consisted of interviews, measurement of anthropometric indices, collection and testing of biologic specimens. The current project, which was done based on data from Afghanistan NNS-2013, was ethically approved at Tehran University of Medical sciences (IR.TUMS.MEDICINE.REC.1399.581). Before participation in the survey, written informed consent was obtained from the head of all selected households.

Several steps were taken to ensure the quality of data collection. The Aga Khan University developed the questionnaire and conducted a pilot study to validate it. The equipment was checked daily before field activity. The representatives of Silk Route Training and Research Organization (SRTRO), Aga Khan University (AKU), UNICEF, and Public Nutrition Department (PND) reviewed the progress of data collection daily. Similar quality assurance was considered during data entry and cleaning.

### Assessment of anthropometric measurements

Height and weight measurements were taken from all survey target age groups (Children 0–59 months, index mother, youngest women of reproductive age, adolescent girls (10–19 years of age) and elderly of above 50 years of age). Measurements of weight and height were conducted for all under five children (0–59 months). Every under-five child present in the household at the time of visit was included in the survey based on acquisition of consent from the parent or caretaker. The youngest child in the household was selected as the index child and measured three times for all height and weight and the mean measurements were documented. Other children and adults were measured one time. Weight and height measurements were made using lightweight SECA scales (with digital screens) designed and manufactured under the authority of the United Nations Children’s Fund (UNICEF). Children under 2 years of age were measured lying down on the board (recumbent length), and standing height was measured for older children.

### Assessment of biochemical measurements

Biochemical samples were collected for assessment of essential micronutrient deficiencies in women of reproductive age (15–49 years), adolescent girls (10 to 19 years) and children (6 to 59 months and 7–12 years of age). Micronutrients were tested to ascertain the levels of various micronutrient deficiencies; hemoglobin, vitamin A, vitamin D, and urinary iodine were measured. Biochemical specimens were analyzed in the Nutrition Research Laboratory of Aga Khan University (AKU), Pakistan. To examine vitamin D status, serum levels of 25-OH-D were measured by electrochemiluminescence protein binding assay. Vitamin A status was examined by measuring serum retinol concentrations by quantitative-high performance liquid chromatography photodiode array detection method. Urinary iodine was measured by inductively coupled plasma-mass spectrometry. Hemoglobin levels were measured using HemoCue machine in Afghanistan. Assessment of micronutrients in different age groups is given in **S2 Table**.

### Inclusion and exclusion criteria

Individuals who had anthropometric measurements and/or biochemical specimens were selected for the current study. At the household level, at least two members who had the anthropometric measurements and/or biochemical specimens were eligible for the study. Pregnant women and those with implausible data were excluded. At the household level, those without data for less than two members were excluded from final analysis.

### Definition of double burden of malnutrition

To define DBM, the deficiency states were defined as follow: 1) Stunting: <3^rd^ percentile of height-for-age based on WHO criteria in 0-19y [18]; 2) Wasting: <3^rd^ percentile (WHO cut-off points) age- and sex-specific weight-for-length for children aged <2 y and <3^rd^ percentile (WHO cut-off points) age- and sex-specific BMI-for-age for children and adolescents aged 2-19y [18]; 3) Underweight: <3^rd^ percentile (WHO cut-off points) age- and sex-specific weight-for-age for children aged 0-10y, <3^rd^ percentile (WHO cut-off points) age- and sex-specific BMI-for-age for adolescents aged >10–19 y and BMI<18.5 kg/m^2^ for those aged >19 y [18,19]. 4) Vitamin D deficiency: <20ng/ml [20]; 5) Anemia: hemoglobin concentrations of less than 11 g/dL among children aged <5.0 years, and less than 11.5 g/dL among those aged ≥5.0, <12.0 years. These cut-off points was less than 12.0 g/dL among those aged≥12.0 and <15.0 years. In non-pregnant females aged ≥ 15.0 years, this cut-off point was 12.0 g/dL [21,22]. 6) Vitamin A deficiency: serum retinol concentrations below a cut-off of 0.70 μmol/L (or 20 μg/dL) [19]. 7) Iodine deficiency: a median UIC of <100 μg/L [23].

In addition, overweight/obese was defined as >85^th^ percentile (WHO cut-off points) age- and sex-specific weight-for-length for children aged <2 y and >85^th^ percentile (WHO cut-off points) age- and sex-specific BMI-for-age for those aged 2–19 y and BMI ≥25 kg/m^2^ for those aged >19 y [18,19]. It is worth mentioning; the term overweight indicates overweight/obese in all parts of this article.

Intra-individual DBM was defined as the co-existence of “overweight” along with “stunting or micronutrient deficiencies” (including anemia, vitamin A deficiency, vitamin D deficiency and iodine deficiency). At the household level, DBM was considered as having at least one household member as overweight and at least one another member of that household as undernourished (stunted, wasted, underweight or any micronutrient deficiency).

### Statistical analysis

For statistical analysis, SPSS and Stata software were used. Characteristics of participants were described as mean±SD, frequency and percentage. To identify the prevalence of DBM at individual level, cross-tabulation was performed in SPSS. To identify, DBM at household level, STATA was used to obtain 95% CIs. To describe DBM at the household level, it was necessary to have data from at least two persons at the same household. If there was data available for one person at household, such households were excluded from the analysis. With regard to serum retinol concentrations and blood hemoglobin levels, adjusted values were used in analysis. Serum retinol concentrations in the dataset were adjusted for C-reactive protein and Alpha-1 acid Glycoproteins. Hemoglobin levels were also adjusted for altitudes.

It is worth mentioning that, 678 individuals aged under 5 y had simultaneously overweight (based on weight for height or BMI for age) and underweight (based on weight for age) because of the use of different definitions in the guidelines. All of them were considered overweight based on weight for height or BMI for age standards.

## Results

**Fig 1:**
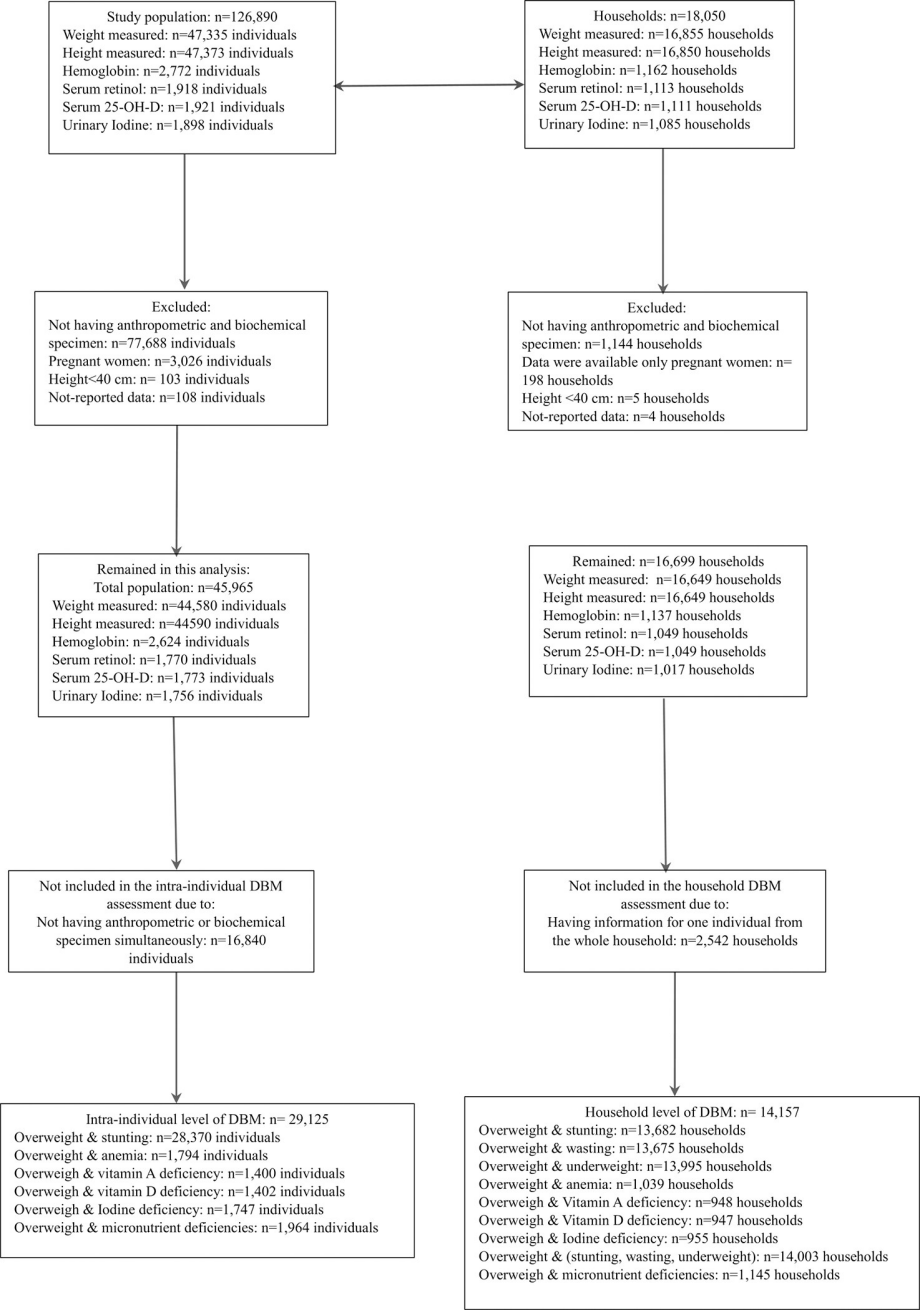
Flowchart of study population.

The number of study population is given in **Fig 1**. The whole survey included 126,890 individuals (64,987 males and 61,903 females) with an average age of 19.3 ±16.8 years. In this population, weight was measured among 47,335 people and height was available for 47373 people. Serum levels of Serum 25-OH-D, Serum retinol and hemoglobin were available for 1921, 1918 and 2772 individuals, respectively. Urinary iodine was measured among 1898 individuals. Therefore, considering all required variables to define DBM, data was available from 29,125 people in intra-individual and 14,157 households for final analysis.

### Characteristics of study participants

**Table 1** shows the characteristics of all the study participants. Mean age of children under five years (n = 22,164) was 2.3±1.4 years (boys: 2.3±1.4 and girls: 2.3±1.4). Their mean BMI was 16.1±3.4 kg/m^2^. Among children under five years (CU5), the mean hemoglobin level was 11.0±1.7g/dL. Most children were living in rural areas (n = 19,174, 86.5%). Mean age of adolescent girls (n = 6,883) was 13.8±2.8 years and their mean BMI was 18.8±3.6 kg/m^2^. The mean of hemoglobin level in this age group was 12.4±1.6 g/dL. Of them 82.4% (n = 5675) were living in rural areas, 8.6% (n = 591) were married and 46.5% (n = 3,204) were illiterate. Mean age of adults (age range: 19 to 65 y) was 36.6±12.3 (men: 55.4±4.5 and women: 34.9±11.3) and their mean BMI was 23.2±4.2 kg/m^2^. Among adults, 85.5% (n = 13,627) were living in rural areas, 90.4% (n = 14,410) were married, and 84.1% (n = 13,409) were illiterate. Examining older adults (aged >65 y), we found mean age of 72.7±6.4 y, of them 80.8% (n = 425) were living in rural areas, 50% (n = 263) were married, and 85% (n = 447) were illiterate. Among older adults, the mean BMI was 22.4±4.4 kg/m^2^.

**Table 1:** Characteristics of study participants ^a^.

	**0–5 y**			**10–19 y**
	**Boys**	**Girls**	**Total**	**Girls**
**Age** ^b^	2.3 ±1.4 (11,368)	2.3 ±1.4 (10,796)	2.3 ±1.4 (22,164)	13.8 ±2.8 (6,883)
**Height** ^b^	80.9 ±14.2 (11,305)	80.2 ±14.2 (10,724)	80.6 ±14.2 (22,029)	144.3 ±12.5 (6,392)
**Weight** ^**b**^	10.7 ±3.4 (11,303)	10.4 ±3.4 (10,714)	10.5 ±3.4) (22,017)	40.0 ±11.8 (6,392)
**BMI** ^**b**^	16.1±3.3 (11,288)	16.0±3.5 (10,703)	16.1±3.4 (21,991)	18.8±3.6 (6,392)
**Hemoglobin** ^b^	11.0 ±1.8 (475)	11.0 ±1.7 (430)	11.0 ±1.7 (905)	12.4 ±1.6 (785)
**Serum retinol** ^b^	20.6 ±8.5 (372)	21.0 ±8.0 (351)	20.8 ±8.2 (723)	30.2 ±9.1 (69)
**Serum 25-OH-D** ^b^	15.9 ±11.7 (375)	14.5 ±9.1 (353)	15.2 ±10.5 (728)	8.0 ±4.4 (69)
**Urinary iodine** ^b^	--	--	--	157.1±120.7 (148)
**Marital status**				
**Married**	--^c^	--	--	8.6 (591)
**Un-married**	--	--	--	91.3 (6,285)
**Widow/widower**	--	--	--	0.1 (4)
**Divorce/separated**	--	--	--	0 (2)
**Not-reported**	--	--	--	0 (1)
**Education**				
**Illiterate**	--	--	--	46.5 (3,204)
**Under-diploma**	--	--	--	51.2 (3,523)
**Diploma**	--	--	--	1.2 (86)
**University**	--	--	--	0.1 (10)
**Not-reported**	--	--	--	0.9 (60)
**Place of residence**				
**Urban**	13.7 (1,556)	13.9 (1,434)	13.5 (2,990)	17.6 (1,208)
**Rural**	86.3 (9,812)	86.1 (9,362)	86.5 (19,174)	82.4 (5,675)

	**19–65 y**			**>65 y**		
	**Males**	**Females**	**Total**	**Males**	**Females**	**Total**
**Age** ^**b**^	55.4 ±4.5 (1,312)	34.9 ±11.3 (14,633)	36.6 ±12.3 (15,945)	73.7 ±7.0 (242)	71.9 ±5.8 (284)	72.7 ±6.4 (526)
**Height** ^**b**^	166.9 ±8.1 (1,312)	155.6 ±6.3 (14,331)	156.6 ±7.2 (15,643)	165.6 ±7.6 (242)	151.6 ±7.1 (248)	158.0 ±10.1 (526)
**Weight** ^**b**^	66.0 ±11.9 (1,312)	56.1 ±10.8 (14,333)	57.0 ±11.3 (15,645)	61.9 ±11.9 (242)	51.5 ±11.8 (248)	56.3 ±12.9 (526)
**BMI** ^**b**^	23.7±3.9 (1,312)	23.2±4.2 (14,331)	23.2±4.2 (15,643)	22.5±3.6 (242)	22.4±5.0 (248)	22.4±4.4 (526)
**Hemoglobin** ^**b**^	--	12.1 ±1.6 (934) ^d^	12.1 ±1.6 (934)	--	--	--
**Serum retinol** ^**b**^	--	33.3 ±12.4 (978) ^d^	33.3 ±12.4 (978)	--	--	--
**Serum 25-OH-D** ^**b**^	--	8.4 ±7.1 (976) ^d^	8.4 ±7.1 (976)	--	--	--
**Urinary iodine** ^**b**^	--	142.6 ±109.6 (926) ^d^	142.6 ±109.6 (926)	--	--	--
**Marital status**						
**Married**	86.4 (1133)	90.7 (13,277)	90.4 (14,410)	65.7 (159)	36.6 (104)	50.0 (263)
**Un-married**	0.7 (9)	2.6 (380)	2.4 (389)	3.3 (8)	6.3 (18)	4.9 (26)
**Widow/widower**	12.5 (164)	6.5 (953)	7.0 (1,117)	31.0 (75)	56.7 (161)	44.9 (236)
**Divorce/separated**	0.2 (2)	0.1 (14)	0.1 (16)	--	0.4 (1)	0.2 (1)
**Not-reported**	0.3 (4)	0.1 (9)	0.1 (13)	--	--	--
**Education**						
**Illiterate**	60.1 (789)	86.2 (12,620)	84.1 (13,409)	73.1 (177)	95.1 (270)	85.0 (447)
**Under-diploma**	28.0 (367)	10.7 (1,559)	12.1 (1,926)	22.3 (54)	3.5 (10)	12.2 (64)
**Diploma**	6.5 (85)	1.6 (233)	2.0 (318)	3.3 (8)	--	1.5 (8)
**University**	4.6 (61)	0.9 (125)	1.2 (186)	0.8 (2)	--	0.4 (2)
**Not-reported**	0.8 (10)	0.7 (96)	0.7 (106)	0.4 (1)	1.4 (4)	1.0 (5)
**Place of residence**						
**Urban**	11.7 (154)	14.8 (2,164)	14.5 (2,318)	15.7 (38)	22.2 (63)	19.2 (101)
**Rural**	88.3 (1,158)	85.2 (12,469)	85.5 (13,627)	84.3 (204)	77.8 (221)	80.8 (425)

^a^ All values are percentages (numbers), unless indicated.

^b^ Values are mean ±SD (numbers).

^c^ Data were not available.

^d^ Data available from 19-50y.

### Prevalence of DBM components

**Table 2** demonstrates the prevalence of components of DBM. Among children under five, we found the prevalence of stunting as 46.2% (95% CI: 45.6; 46.9). In addition, 11.6% of them were wasted, 24.4% were underweight and 19.9% were overweight or obese. The prevalence of anemia, vitamin D and vitamin A deficiency among them was 44.8, 80.6 and 50.5%, respectively.

**Table 2:** Prevalence of DBM components ^a^.

	**0–5 y**			**10–19 y**	**19–65 y**		
	**Boys Girls Total**			**Girls**	**Males Females Total**		
**Overweight**							
**N**	11,285	10,693	21,978	6,392	1,312	14,331	15,643
	20.1 (19.4; 20.9)	19.7 (18.9; 20.4)	19.9 (19.4; 20.4)	10.4 (9.7; 11.2)	33.1 (30.5;35.6)	26.6 (25.8; 27.3)	27.1 (26.4; 27.8)
**Stunting**							
**N**	11,305	10,724	22,029	6392			
	48.0 (47.1; 48.9)	44.4 (43.5; 45.30	46.2 (45.6; 46.90)	35.3 (34.1; 36.4)	--^b^	--	--
**Wasting**							
**N**	11,279	10,693	21,972	6,392			
	12.5 (11.9; 13.1)	10.5 (10; 11.1)	11.6 (11.1; 12.0)	8.7 (8.0; 9.4)	--	--	--
**Underweight**							
**N**	11,303	10,714	22,017	6,392	1,312	14,331	15,643
	25.6 (24.8; 26.4)	23.1 (22.3; 23.9)	24.4 (23.8; 25.0)	10.6 (9.9; 11.4)	6.3 (5.0; 7.7)	9.4 (8.9; 9.9)	9.1 (8.7; 9.6)
**Anemia**							
**N**	475	430	905	785		934	934
	42.9 (38.5; 47.5)	46.7 (42.1; 51.5)	44.8 (41.5; 48.0)	29.3 (26.2; 32.6)	--	41.4 (38.3; 44.6)^c^	41.4 (38.3; 44.6)
**Vitamin D deficiency**							
**N**	375	353	728	69		976	976
	77.3 (72.8; 81.3)	84.1 (79.9; 87.6)	80.6 (77.6; 83.3)	98.6 (90.0; 99.8)	--	95.0 (93.4; 96.2)^c^	95.0 (93.4; 96.2)
**Vitamin A deficiency**							
**N**	372	351	723	69		978	978
	51.6 (46.5; 56.7)	49.3 (44.1; 54.5)	50.5 (46.8; 54.1)	13.0 (6.8; 23.5)	--	11.0 (9.2; 13.2)^c^	11.0 (9.2; 13.2)

	**>65 y**			**All age groups**		
	**Males Females Total**			**Males Females Total**		
**Overweight**						
**N**	242	284	526	12,839	31,700	44,539
	21.1 (16.4; 26.7)	25.4 (20.8; 30.8)	23.4 (20.0; 27.2)	21.5 (20.8; 22.2)	21.0 (20.5; 21.4)	21.1 (20.7; 21.5)
**Stunting**						
**N**				11,305	17,116	28,421
	--	--	--	48.0 (47.1; 48.9)	41.0 (40.3; 41.7)	43.8 (43.2; 44.3)
**Wasting**						
**N**				11,279	17,085	28,364
	--	--	--	12.5 (11.9; 13.1)	9.8 (9.4; 10.3)	10.9 (10.6; 11.3)
**Underweight**						
**N**	242	284	526	12,857	31,721	44,578
	9.1 (5.8; 13.4)	23.2 (18.7; 28.5)	16.7 (13.8; 20.2)	23.3 (22.6; 24.1)	14.4 (14.0; 14.8)	17.0 (16.6; 17.3)
**Anemia**						
**N**				475	2,149	2624
	--	--	--	42.9 (38.5; 47.5)	38.1 (36; 40.1)	38.9 (37.1; 40.8)
**Vitamin D deficiency**						
**N**				375	1,398	1,773
	--	--	--	77.3 (72.8; 81.3)	92.4 (91.0; 93.8)	89.2 (87.7; 90.6)
**Vitamin A deficiency**						
**N**				372	1,398	1,770
	--	--	--	51.6 (46.5; 56.7)	20.7 (18.7; 23.0)	27.2 (25.2; 29.4)

^a^ DBM: Double Burden of Malnutrition; all values are percentages (95% CIs); **Overweight**: >85^th^ percentile (WHO cut-off points) age- and sex-specific weight-for-length for children aged <2 y and >85^th^ percentile (WHO cut-off points) age- and sex-specific BMI-for-age for those aged 2–19 y and BMI ≥25 kg/m^2^ for those aged >19 y; **Stunting**: <3^rd^ percentile of height-for-age (WHO cut-off points) for individuals aged 0–19 y. **Wasting:** <3^rd^ percentile (WHO cut-off points) age- and sex-specific weight-for-length for children aged <2 y and <3^rd^ percentile (WHO cut-off points) age- and sex-specific BMI-for-age for children and adolescents aged 2-19y; **Underweight**: <3^rd^ percentile (WHO cut-off points) age- and sex-specific weight-for-age for children 0–10 y, <3^rd^ percentile (WHO cut-off points) age- and sex-specific BMI-for-age adolescents aged >10–19 y and BMI<18.5 kg/m^2^ for those aged >19 y; **Anemia**: Defined as hemoglobin concentrations of less than 11 g/dL among children aged <5.0 years, and less than 11.5 g/dL among those aged ≥5.0- <12.0 years. The corresponding cut-off points for those aged ≥12.0- <15.0 years and non-pregnant females aged ≥15.0 years was less than 12 g/dL; **Vitamin D deficiency**: Serum 25-OH-D levels of <20ng/mL; **Vitamin A deficiency**: Serum retinol concentrations of <0.70 μmol/L (or 20 μg/dL).

^b^ Data were not available.

^c^ Due to having biochemical specimen from 19-50y, these deficiencies presented only for 19–50 y.

Among adolescent girls, we found the prevalence of stunting as 35.3% (95% CI: 34.1; 36.4). In total, 8.7% (8.0; 9.4) were wasted, 10.6% (9.9; 11.4) were underweight and 10.4% were overweight or obese. The prevalence of anemia, vitamin D and vitamin A deficiency among them was 29.3, 98.6, and 13%, respectively. Overall, 37.8% of adolescent girls had iodine deficiency.

Among adults aged 19-65y, 9.1% were underweight, while 27.1% were overweight. Of them, 41.4% were anemic, 95% had vitamin D deficiency, 11% had vitamin A deficiency and 40.4% had iodine deficiency. Among adults aged over 65 years, the prevalence of underweight and overweight was 16.7% (95%CI: 13.5; 20.2) and 23.4% (95%CI: 20.0; 27.2), respectively.

Combining all age groups, we found that 43.8% of study population had stunting, 10.9% were wasted, 17.0% were underweight and 21.1% were overweight. The prevalence of anemia, vitamin D and vitamin A deficiency among them was 38.9, 89.2 and 27.2%, respectively. A totally of, 35.8% had iodine deficiency.

It is noteworthy that urinary iodine was measured among children aged 7–12 year. Iodine deficiency was prevalent among 29.4% of children aged 7–12 year (Boys: 29.4; 95% CI: 25.1; 33.9 and Girls: 29.3; 95% CI: 24.5; 34.1).

**Fig 2:**
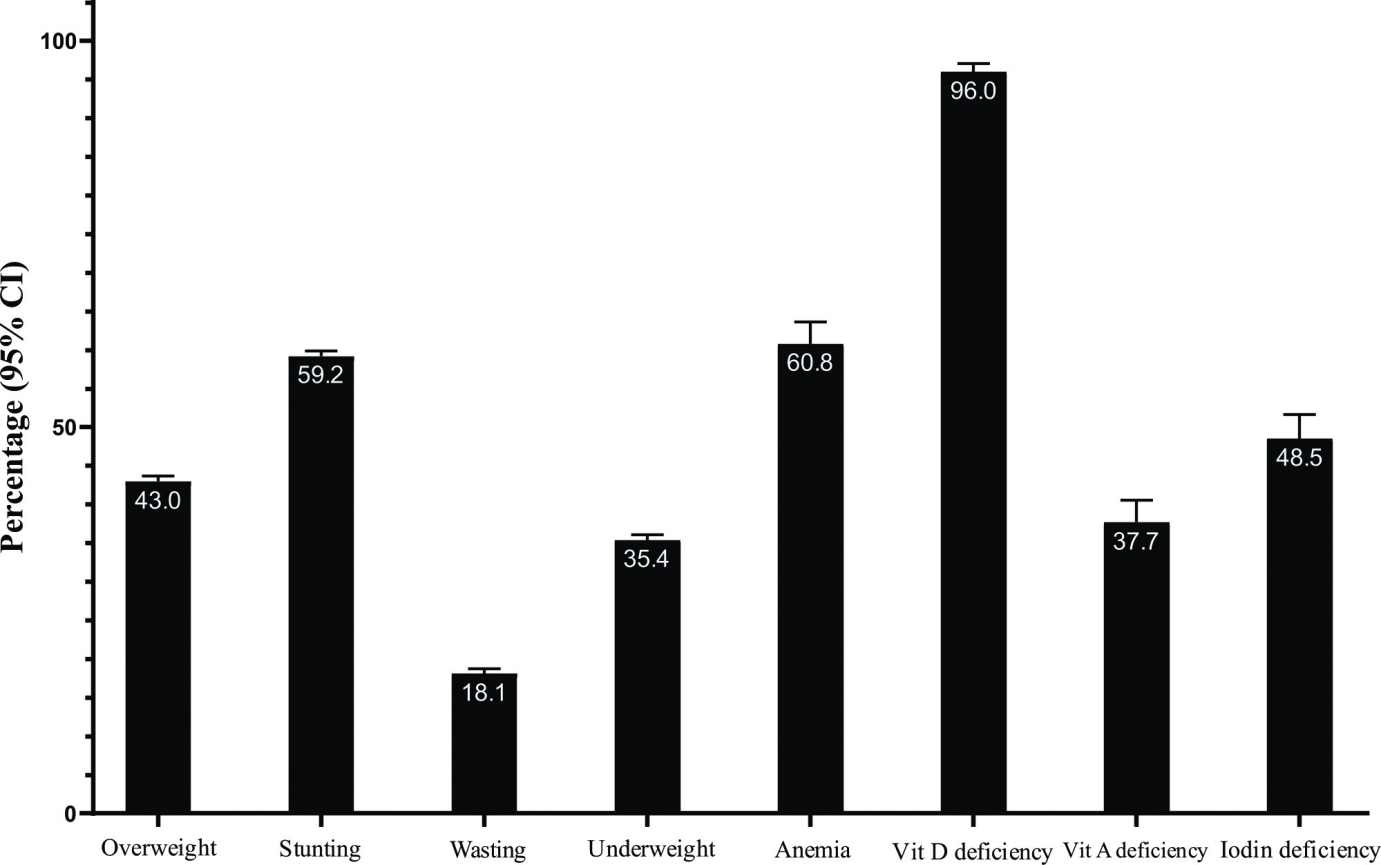
Prevalence of DBM components at household level. Overweight: >85^th^ percentile (WHO cut-off points) age- and sex-specific weight-for-length for children aged <2 y and >85^th^ percentile (WHO cut-off points) age- and sex-specific BMI-for-age for those aged 2–19 y and BMI ≥25 kg/m^2^ for those aged >19 y; Stunting: <3^rd^ percentile of height-for-age (WHO cut-off points) for individuals aged 0–19 y. Wasting: <3^rd^ percentile (WHO cut-off points) age- and sex-specific weight-for-length for children aged <2 y and <3^rd^ percentile (WHO cut-off points) age- and sex-specific BMI-for-age for children and adolescents aged 2-19y; Underweight: <3^rd^ percentile (WHO cut-off points) age- and sex-specific weight-for-age for children 0–10 y, <3^rd^ percentile (WHO cut-off points) age- and sex-specific BMI-for-age adolescents aged >10–19 y and BMI<18.5 kg/m^2^ for those aged >19 y; Anemia: Defined as serum hemoglobin concentrations of less than 11 g/dL among children aged <5.0 years, and less than 11.5 g/dL among those aged ≥5.0- <12.0 years. The corresponding cut-off points for those aged ≥12.0- <15.0 years and non-pregnant females aged ≥15.0 years was less than 12 g/dL. Vitamin D deficiency: Serum 25-OH-D levels of <20ng/mL; Vitamin A deficiency: Serum retinol concentrations of <0.70 μmol/L (or 20 μg/dL). Iodine deficiency: A median urinary iodine concentration of <100 μg/L.

Prevalence of DBM components at household is provided in **Fig 2**. The prevalence of overweight among households was 43.0% (95% CI: 42.2; 43.7. Stunting and wasting was prevalent among 59.2% (58.4; 59.9) and 18.1% (17.5; 18.7) of households, respectively. In addition, 35.4% (34.6; 36.1) of households were suffering from underweight, 60.8% (57.9; 63.6) from anemia and 96% (94.6; 97.1) from vitamin D deficiency. In total, vitamin A deficiency was prevalent in 37.7% (34.8; 40.6) of households and iodine deficiency among 48.5% (45.4; 51.6).

### Prevalence of intra-individual level of DBM

Prevalence of intra-individual level of DBM are provided in **Table 3**. The overall prevalence of intra-individual DBM in the whole study population was 12.5% (95% CI: 12.1; 12.9). We examined co-existence of overweight and individual under-nutrition states and found that 11.7% (11.3; 12.1) of study population had overweight and stunting, 9.5% (8.2; 11.0) had overweight and anemia and 24.4% (22.2; 26.7) had overweight and vitamin D deficiency. Furthermore, 5.6% (4.4; 6.9) of study participants had overweight alongside vitamin A deficiency, while 13.4% (11.1; 16.0) had overweight and iodine deficiency simultaneously. Overweight along with a combination of micronutrient deficiencies was seen in 20.5% (18.8; 22.4) of whole study population.

**Table 3:** Prevalence of intra-individual level of DBM in all study participants and separately by age groups ^a^.

	0-5y						10–19 y		19–65 y ^b^		All age groups					
	Boys		Girls		Total		Girls		Females		Males		Females		Total	
	Overweight		Overweight		Overweight		Overweight		Overweight		Overweight		Overweight		Overweight	
	No	Yes	No	Yes	No	Yes	No	Yes	No	Yes	No	Yes	No	Yes	No	Yes
**Under-nutrition**																
**No**	44.7	5.4	47.6	5.9	46.1	5.7	56.5	6.5	2.6	1.5	44.7	5.4	48.9	5.9	47.3	5.7
**Yes**	35.2	**14.8**	32.7	**13.7**	34.0	**14.3**	33.1	**3.9**	62.6	**33.2**	35.2	**14.8**	34.1	**11.0**	34.5	**12.5**
**Stunting**																
**No**	46.4	5.6	49.3	6.3	47.8	5.9	58.1	6.6	--^c^	--	46.4	5.6	52.6	6.4	50.1	6.1
**Yes**	33.5	**14.5**	31.0	**13.4**	32.3	**14.0**	31.4	**3.8**	--	--	33.5	**14.5**	31.2	**9.8**	32.1	**11.7**
**Anemia**																
**No**	48.5	10.1	41.8	10.8	45.3	10.4	62.1	9.1	36.6	20.7	48.5	10.1	44.0	15.3	45.0	14.0
**Yes**	34.7	**6.8**	36.8	**10.6**	35.7	**8.6**	26.8	**1.9**	28.4	**14.3**	34.7	**6.8**	30.4	**10.4**	31.4	**9.5**
**Vitamin D deficiency**																	
**No**	19.4	3.2	13.5	2.6	16.6	2.9	2.4	0.0	3.2	1.6	19.4	3.2	6.2	1.8	9.4	2.1
**Yes**	62.9	**14.4**	67.0	**17.0**	64.9	**15.6**	80.5	**17.1**	62.3	**32.9**	62.9	**14.4**	64.4	**27.6**	64.1	**24.4**
**Vitamin A deficiency**																	
**No**	38.2	9.5	38.8	10.7	38.5	10.0	73.2	14.6	57.3	31.6	38.2	9.5	52.5	24.9	49.1	21.1
**Yes**	44.1	**8.3**	41.4	**9.1**	42.8	**8.7**	9.8	**2.4**	8.1	**2.9**	44.1	**8.3**	17.9	**4.7**	24.2	**5.6**
**Iodine deficiency**																
**No**	--	--	--	--	--	--	51.3	8.8	39.6	20.2	--	--	40.8	19.0	40.8	19.0
**Yes**	--	--	--	--	--	--	36.3	**3.8**	25.6	**14.5**	--	--	26.8	**13.4**	26.8	**13.4**
**Total micronutrient** ^**d**^ **deficiency**																
**No**	15.9	2.2	12.2	1.7	14.1	2.0	52.9	6.9	2.6	1.5	15.9	2.2	16.7	2.8	16.5	2.6
**Yes**	67.8	**14.1**	67.8	**18.3**	67.8	**16.1**	34.4	**3.8**	62.6	**33.2**	67.8	**14.8**	58.0	**22.4**	60.3	**20.5**

^a^ Double Burden of Malnutrition (DBM): Overweight along with under nutrition (stunting or anemia or vitamin D deficiency or vitamin A deficiency or iodine deficiency).

Overweight: >85th percentile (WHO cut-off points) age- and sex-specific weight-for-length for childre018n aged <2 y and >85th percentile (WHO cut-off points) age- and sex-specific BMI-for-age for those aged 2–19 y and BMI ≥25 kg/m2 for those aged >19 y; Stunting: <3rd percentile of height-for-age (WHO cut-off points) for individuals aged 0–19 y; Anemia: Defined as hemoglobin concentrations of less than 11 g/dL among children aged <5.0 years, and less than 11.5 g/dL among those aged ≥5.0- <12.0 years. The corresponding cut-off points for those aged ≥12.0- <15.0 years and non-pregnant females aged ≥15.0 years was less than 12 g/dL.; Vitamin D deficiency: Serum 25-OH-D levels of <20ng/mL; Vitamin A deficiency: Serum retinol concentrations of <0.70 μmol/L (or 20 μg/dL). Iodine deficiency: A median urinary iodine concentration of <100 μg/L.

^b^ Due to having biochemical specimen from 19–50 y, DBM is defined from 19–50 y.

^c^ Data were not available.

^d^ Total micronutrient deficiency: Anemia or vitamin D deficiency or vitamin A deficiency or iodine deficiency. All values are percentage.

The whole prevalence of intra-individual DBM among CU5 was 14.3% (boys: 14.8% and girls: 13.7%). Prevalence of co-occurrence of overweight and individual under-nutrition states among CU5 were as follows: In total, 14.0% (95% CI: 13.5; 14.5) of CU5 were overweight and stunted. Having overweight and anemia was prevalent among 8.6% (6.7; 10.7) of CU5, while overweight and vitamin D deficiency was among 15.6% (13.0; 18.6). Totally, 8.7% (6.6; 11.1) of CU5 were overweight and had simultaneously vitamin A deficiency. The co-prevalence of overweight and combination of micronutrient deficiencies was seen among 16.1% (13.8; 18.7) of CU5.

Among adolescent girls, the whole prevalence of intra-individual DBM was 3.9% (95% CI: 3.5; 4.4). In total, 3.8% (3.4; 4.3) of adolescent girls were overweight and stunted, 1.9% (0.7; 4.1) had overweight and anemia simultaneously, 17.1% (8.1; 32.5) had overweight and vitamin D deficiency together and 2.4% (0.6; 12.6) had overweight and vitamin A deficiency. Simultaneous overweight and iodine deficiency existed in 3.8% (0.8; 10.6) of adolescents. Totally, the prevalence of overweight along with a combination of micronutrient deficiencies was seen in 3.8% (2.0; 6.3) of adolescent girls.

Examining adults aged 19–65 y, we found that intra-individual DBM was prevalent among 33.2% (95% CI: 30.0; 36.7); such that 14.3% (11.8; 17.1) of them were suffering from overweight and anemia, 32.9% (29.5; 36.3) from overweight and vitamin D deficiency, and 2.9% (1.8; 4.5) from overweight and vitamin A deficiency. Combination of overweight and iodine deficiency was seen among 14.5% (12.1; 17.4) of this population.

### Prevalence of household level of DBM

**Fig 3:**
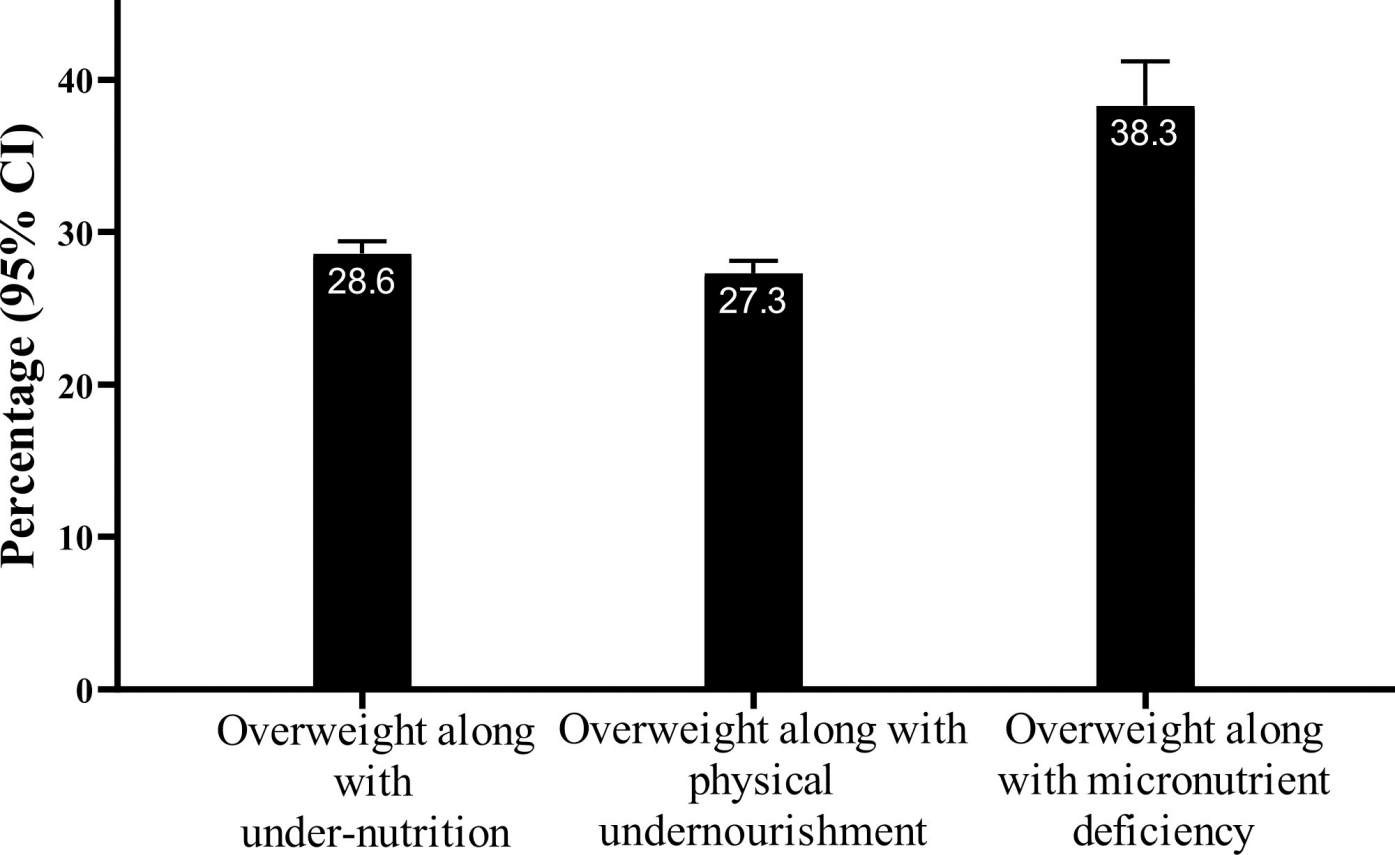
Prevalence of DBM at household level. Overweight: >85^th^ percentile (WHO cut-off points) age- and sex-specific weight-for-length for children aged <2 y and >85^th^ percentile (WHO cut-off points) age- and sex-specific BMI-for-age for those aged 2–19 y and BMI ≥25 kg/m^2^ for those aged >19 y; Stunting: <3^rd^ percentile of height-for-age (WHO cut-off points) for individuals aged 0–19 y. Wasting: <3^rd^ percentile (WHO cut-off points) age- and sex-specific weight-for-length for children aged <2 y and <3^rd^ percentile (WHO cut-off points) age- and sex-specific BMI-for-age for children and adolescents aged 2-19y; Underweight: <3^rd^ percentile (WHO cut-off points) age- and sex-specific weight-for-age for children 0–10 y, <3^rd^ percentile (WHO cut-off points) age- and sex-specific BMI-for-age adolescents aged >10–19 y and BMI<18.5 kg/m^2^ for those aged >19 y; Anemia: Defined as serum hemoglobin concentrations of less than 11 g/dL among children aged <5.0 years, and less than 11.5 g/dL among those aged ≥5.0- <12.0 years. The corresponding cut-off points for those aged ≥12.0- <15.0 years and non-pregnant females aged ≥15.0 years was less than 12 g/dL. Among men aged ≥15.0 years, the cut-off point of 13.0 g/dL was used; Vitamin D deficiency: Serum 25-OH-D levels of <20ng/mL; Vitamin A deficiency: Serum retinol concentrations of <0.70 μmol/L (or 20 μg/dL). Iodine deficiency: A median urinary iodine concentration of <100 μg/L. Under-nutrition: Stunting or wasting or underweight or anemia or vitamin A deficiency or vitamin D deficiency or iodine deficiency. Physical undernourishment: Stunting or wasting or underweight. Micronutrient deficiency: Anemia or vitamin A deficiency or vitamin D deficiency or iodine deficiency.

Prevalence of household level of DBM is provided in **Fig 3**. The whole prevalence of DBM at household level was 28.6% (95% CI: 27.9; 29.4). However, examining overweight along with a combination of physical undernourishment (stunting or wasting or underweight) at the household level, we found that 27.3% (26.6; 28.1) of households were affected. Overweight and combination of micronutrient deficiencies was prevalent among 38.3% (35.5; 41.2) of households.

**Fig 4:**
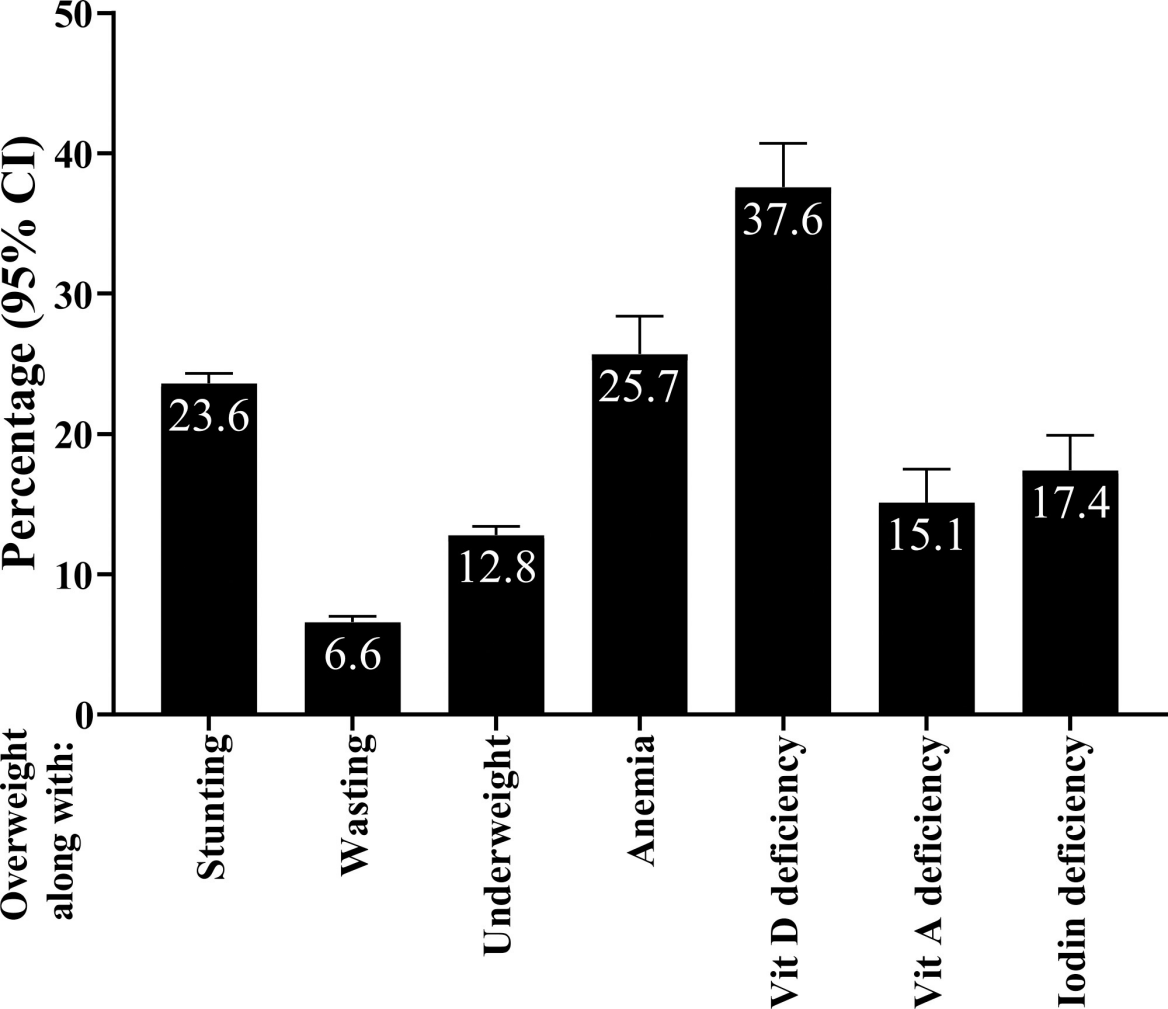
Prevalence of DBM at household level based on individual types of under-nutrition. Overweight: >85^th^ percentile (WHO cut-off points) age- and sex-specific weight-for-length for children aged <2 y and >85^th^ percentile (WHO cut-off points) age- and sex-specific BMI-for-age for those aged 2–19 y and BMI ≥25 kg/m^2^ for those aged >19 y; Stunting: <3^rd^ percentile of height-for-age (WHO cut-off points) for individuals aged 0–19 y. Wasting: <3^rd^ percentile (WHO cut-off points) age- and sex-specific weight-for-length for children aged <2 y and <3^rd^ percentile (WHO cut-off points) age- and sex-specific BMI-for-age for children and adolescents aged 2-19y; Underweight: <3^rd^ percentile (WHO cut-off points) age- and sex-specific weight-for-age for children 0–10 y, <3^rd^ percentile (WHO cut-off points) age- and sex-specific BMI-for-age adolescents aged >10–19 y and BMI<18.5 kg/m^2^ for those aged >19 y; Anemia: Defined as serum hemoglobin concentrations of less than 11 g/dL among children aged <5.0 years, and less than 11.5 g/dL among those aged ≥5.0- <12.0 years. The corresponding cut-off points for those aged ≥12.0- <15.0 years and non-pregnant females aged ≥15.0 years was less than 12 g/dL. Vitamin D deficiency: Serum 25-OH-D levels of <20ng/mL; Vitamin A deficiency: Serum retinol concentrations of <0.70 μmol/L (or 20 μg/dL). Iodine deficiency: A median urinary iodine concentration of <100 μg/L.

**Fig 4** presents prevalence of DBM at the household level, as defined by the combination of overweight and individual states of under-nutrition. In total, 23.6% (22.9; 24.3) of households had overweight and stunting together. Overweight and wasting was prevalent among 6.6% (6.2; 7.0) of households. Coexistence of overweight and underweight was seen among 12.8% (12.3; 13.4) and overweight and anemia among 25.7% (23.0; 28.4) of households. The household magnitude of overweight and vitamin D deficiency was seen among 37.6% (34.6; 40.7) and overweight and vitamin A deficiency among 15.1% (12.9; 17.5). In total, 17.4% (15.1; 19.9). of households were affected by overweight and iodine deficiency simultaneously.

## Discussion

In this cross sectional study, based on data from Afghanistan national nutrition survey, the magnitude of double burden of malnutrition at intra-individual and household level was assessed. Overall, the prevalence of intra-individual and household level of DBM was 12.5% and 28.6%, respectively. It is worth mentioning that this is the first comprehensive study in Afghanistan that examined the magnitude of intra-individual and household level of DBM.

Nutrition transition, changing dietary patterns and lifestyle are occurring in developing countries. Not only under-nutrition but also over-nutrition is prevalent in these countries. Focusing on one side of malnutrition would be harmful and might affect the health of forgotten group. Coexistence of both sides of malnutrition is a big challenge for policy makers to overcome these problems. Therefore, having a precise estimation at national and provincial level is the first step for policy makers to consider the problem and make policies properly.

Among all study populations, overweight was more prevalent among adults aged 19-65y and males than females. Looking at sub-types of physical undernourishment, the magnitude of stunting was higher than the rest. In terms of micronutrient deficiency, vitamin D deficiency was more prevalent in all age groups and gender. In addition, vitamin A deficiency was less prevalent compared to other forms. The prevalence of vitamin D deficiency among different age groups and gender ranged from 22.0 to 81.1% in Egypt, Saudi Arabia, India, United Arabic Emirates, Iran, and Pakistan [24–29]. To illustrate, 75.1% of women and 72.1% of men in Iran and 22.0% of boys and 49.5% of girls in Saudi Arabia suffered from vitamin D deficiency [25,27]. In addition, among these countries, Pakistan showed a higher prevalence of vitamin D deficiency which is comparable to our findings [26]. Moreover, the prevalence of vitamin A deficiency in Pakistan and India is 31.8% and 19.0%, respectively [24,26]. These findings demonstrate that the prevalence of vitamin A deficiency in our study is less than in Pakistan and higher than in India. In addition, studies from Ethiopia and Pakistan show the magnitude of anemia as 53.6% in Pakistan and 34.4% in Ethiopia [26,30]. The magnitude of iodine deficiency was 17.0% in India, which is lower than our findings [24].

In this study, we found that 12.5% of individuals in Afghanistan are affected simultaneously by a combination of under- and over-nutrition. In other words, these individuals suffered from a state of under-nutrition (including stunting, or a vitamin or mineral deficiency) and at the same time there were overweight. Intra-individual level of DMB was examined in different age group and gender. The coexistence of overweight and micronutrient deficiencies was more common in adults aged 19-65y. Examining the individual types of micronutrient deficiencies and overweight, we found that the co-occurrence of overweight and vitamin D deficiency was more prevalent in both genders in all age groups. Moreover, overweight alongside stunting was observed in a higher ratio at CU5 and boys compared to other groups. Comparing findings of current study with adjacent geographic including Pakistan, Iran, and Tajikistan, reports demonstrated that different forms of malnutrition exist in these areas [31–33]. To illustrate, 0.9–4.0% of preschool children and 13.4–34.0% of women of reproductive age suffer from different types of DBM at individual levels [33,34]. In addition, DBM was 3.93% prevalent in households in Pakistan [16]. Considering the nutrition transition in Iran and Tajikistan, overweight/obesity is increasing while undernutrition is still prevalent as well [31,32,35]. No report is available about the coexistence of over and undernutrition at individual and household levels in these countries.

Assessing the magnitude of DBM at intra-individual level is not new in the world. Several other studies from low to middle income countries have examined this problem. Reports from Indonesia, Vietnam, Cambodia, Uruguay, Guatemalan, Mexico and Kenya revealed that the prevalence of intra-individual DBM in these countries ranged from 1.0% to 19.0% [4,36–41]. Comparing the prevalence of intra-individual DBM in our study with earlier ones indicated that the prevalence in Afghanistan was lower than that reported from Kenya and higher than that reported from Mexico [36,37]. However, it must be kept in mind that most of earlier studies have focused on a specific age group or gender, while our estimation was based on all age groups and both gender using a nationally representative data. In addition, the discrepant definitions used for intra-individual DBM in different studies make the cross-study comparisons very difficult and complex. Most of earlier studies have not considered all states of under-nutrition (i.e. stunting and vitamin or mineral deficiencies) at the same time, rather they have considered under-nutrition states individually. For instance, the prevalence reported from Mexico was based on overweight along with stunting or anemia and the one from Indonesia was based on overweight and stunting [37,39]. We considered all levels of under-nutrition together and the estimate we provided was based on having one under-nutrition states along with overweight.

In a pooled analysis of data from 17 national surveys on women of reproductive age, including Afghanistan, Williams et al, reported the prevalence of intra-individual DBM (overweight along with anemia) at the range of 1.0%-18.6% (median: 8.6%). Considering overweight and micronutrient deficiencies together among women of reproductive age, they reported that 1.6%-39.2% (median: 21.9%) of these women were affected. However, they included data only from 568 Afghani women of reproductive age in their study [34]. Engle-Stone et al, reported findings from another pooled analysis based on information from 21 countries, including Afghanistan, on pre-school children. They found that 0% to 9.7% (median: 2.6%) of individuals who were overweight had micronutrient deficiency as well. Furthermore, 0% to 5.0% (median: 1.4%) of individuals had overweight and anemia at the same time. Engle-Stone et al used data from 585 pre-school Afghani children in their study, who had information on serum micronutrient levels [33]. They did not consider stunting in this age group as a state of under-nutrition in their definition of DBM. Therefore, their estimation was based on the combination of overweight and micronutrient deficiencies alone. As we considered all states of under-nutrition in our analysis, which was based on a large sample size of all age groups from either gender, our findings are more comprehensive than previous publications in this regard.

In the present study, we found that the household level of DBM was prevalent among 28.6% of Afghani households. We defined household level of DBM in the current study as overweight along with at least one state of under-nutrition (wasting, underweight, stunting and micronutrient deficiencies) in two different members of the household. We revealed that the magnitude of DBM at the household level was higher compared to the individual level. At the household level, overweight alongside physical undernourishment or micronutrient deficiencies demonstrated that the overweight and micronutrient deficiencies were more prevalent. Altogether, the coexistence of overweight with stunting and overweight with vitamin D deficiency were observed at a higher level than the other combinations.

Comparing our findings with other studies in the world indicated that our estimation for household DBM in Afghanistan was comparable to that reported from Indonesia and it was higher than the estimates reported from Uruguay and Philippine and lower than that reported from Bangladesh [40,42–44]. Overall, examining developing nations in Asia, the prevalence of household level of DBM was reported at the range of 1.54 to 51.7% of households [16,42,44]. Based on data from Latin American countries like Brazil, Uruguay, Mexico, Ecuador and Guatemalan, the prevalence of DBM was reported as 2.6 to 20% of households [4,37,40,45,46]. Examining this issue at African countries, investigators from Kenya showed that 0.6 to 17.3% of households had DBM, as defined by the coexistence of overweight/obese adult and an undernourished child [36]. Most published studies about household DBM had considered a pair of mother-child in their definition, while we considered household DBM as having a state of over-nutrition (overweight) in one member of the household and simultaneously having a state of under-nutrition (wasting, underweight, stunting and micronutrient deficiencies) in one another. Therefore, this different definition should be considered when interpreting our findings. In addition, most studies did not consider a comprehensive assessment of under-nutrition states and most of them have focused on one or two states of under-nutrition only. Overall, the magnitude of household DBM in most under-developed and developing nations, including Afghanistan, is huge and should be considered by policy makers of health system to reduce the burden of this problem to the society.

These findings highlight the complexity of the problem and multiple dimensions of nutrition in Afghanistan. Concomitant high prevalence of overweight and micronutrient deficiencies indicates high consumption of calorie-dense foods among this population. The coexistence of overweight and stunting might be explained by the possibility of overfeeding in children who suffer from stunting as a result of the focus of their parents on their dietary intake. On the other hand, the coexistence of overweight and under-nutrition in the same household demonstrates unhealthy food choices or even food shortages across the household. Another possible reason is that extended families are common in Afghanistan; suggesting that some household members might eat adequately while others might not have enough food to eat. Poverty and income inequality could trigger and worsen this phenomenon. It means that higher prices of healthy foods and lower income could force families to consume unexpansive and unhealthy food choices. Poverty leads to concentrating more on gut hunger rather than cellular hunger (hidden hunger), and thus, some nutrients might be consumed inadequately. Finally, the lack of public awareness about nutrition could be another important reason for the existence of DBM.

This study provides information for the policymakers in the government and international organizations to consider both side of malnutrition in Afghanistan. The implications of these findings would be developing appropriate national macro-policies and strategies and designing appropriate programs to curb the problem inside the country and its implication for the whole Afghani people would be consumption of nutrient-dense foods in their usual diet. In addition, efforts should be done to increase people’s awareness through media, school and health centers about healthy and nutrient-dense foods and balanced diet. Additional strategies including subsidization for healthy foods and increasing the tax for unhealthy foods along with school lunch programs, supporting poor families through food assist programs might be required. Launching food fortification and dietary supplementation can also be further strategies in this country to decrease the burden of DBM.

This study has several strengths. To the best of our knowledge, this the first comprehensive study that assessed the prevalence of DBM from Middle Eastern countries. It is based on a nationally representative sample of Afghani people with a large sample size. In this survey, data were collected from all provinces. In addition to anthropometric measurements, biochemical specimen was also used to identify micronutrient deficiencies at different age groups. Comprehensive definition of under-nutrition in the context of DBM must also be taken into account. Considering DBM at both individual and household level is also another strength. Despite the above-mentioned strengths, our findings should be interpreted while considering some limitations. Information about anthropometric measures and biochemical specimens were not available for all study participants. In addition, data were not available at some age groups. Furthermore, cross-study comparisons were done in the current study while different studies had used different definitions for DBM. As in some households, data had been collected for one person only, we were not able to include these households in our analysis on household DBM. In addition, in some cases, data for anthropometric measures and biochemical specimens were not available for the same individual or even household at the same time.

## Conclusion

This study demonstrated a high prevalence of DBM at individual and household level in Afghanistan. Unlike the public assumption, it seems that both spectrums of malnutrition are prevalent in this country. Therefore, developing appropriate national macro-policies and strategies and designing appropriate programs such as public awareness programs, subsidization, food assistance programs, food fortification and dietary supplementation should be implemented by the ministry of public health, inter- related organs and international health agencies to reduce the burden of this problem in this country.

## Supporting information

S1 Table Description of study population in Afghanistan NNS-2013. (DOCX)

S2 Table List of micronutrient assessment and target groups. (DOCX)
