# Supplementary material for: Double burden of malnutrition in Afghanistan: Secondary analysis of a national survey
Source: PLoS One. 2023 May 30;18(5):e0284952. doi: 10.1371/journal.pone.0284952 (PMC10228760; doi:10.1371/journal.pone.0284952)
Supplement: S1 Table — (DOCX) [file pone.0284952.s001.docx]

**S1 Table. Description of study population in Afghanistan NNS-2013.**

| **Target Group of Anthropometry** | **Measurements** |
| --- | --- |
| All children 0-59 months | Height, Weight |
| Index Mother (Mother of the youngest under five) | Height, Weight |
| Youngest women of reproductive age (15-49) | Height, Weight |
| Adolescent girls (10-19) | Height, Weight |
| Elderly (men & women ≥50 years age) | Height, Weight |
| **Target Group of Biochemical Samples** | **Biochemical Test** |
| Children (6-59 months) | Blood Sample |
| Women (15-49 years) | Blood & Urine Sample |
| Adolescent girls (10-19) | Blood & Urine Sample |
| Children 7-12 years | Urine Sample |

Taken from NNS 2013 report [1].

**Reference**

1. UNICEF. Afghanistan National Nutrition Survey 2013 Survey Report. Dateset) United Nations International Children’s Emergency Fund, Geneva. 2014.
