# Supplementary material for: Double burden of malnutrition in Afghanistan: Secondary analysis of a national survey
Source: PLoS One. 2023 May 30;18(5):e0284952. doi: 10.1371/journal.pone.0284952 (PMC10228760; doi:10.1371/journal.pone.0284952)
Supplement: S2 Table — (DOCX) [file pone.0284952.s002.docx]

**S2 Table. List of micronutrient assessment and target groups.**

| **Micronutrient Assessment** | **Women**  **(19-49 years)** | **Children**  **(6-59 months)** | **Adolescent Girls ^a^ (10-19 years)** | | **Children (7-12 years)** |
| --- | --- | --- | --- | --- | --- |
| **Hemoglobin level** | Yes | Yes | Yes | -- | |
| **Serum retinol** | Yes | Yes | Yes | -- | |
| **Serum 25-OH-D** | Yes | Yes | Yes | -- | |
| **Urinary iodine excretion** | Yes | -- | Yes | Yes | |

^a^ serum retinol and 25-OH-D were collected from 15-19 years, urinary iodine were collected from 10-12y and 15-19y
